# The Risk of Breast Cancer between Western and Mediterranean Dietary Patterns

**DOI:** 10.3390/nu15092057

**Published:** 2023-04-25

**Authors:** Hsueh-Han Tsai, Jyh-Cherng Yu, Huan-Ming Hsu, Chi-Hong Chu, Tzu-Ming Chang, Zhi-Jie Hong, An-Chieh Feng, Chun-Yu Fu, Kuo-Feng Hsu, Ming-Shen Dai, Guo-Shiou Liao

**Affiliations:** 1 Division of General Surgery, Department of Surgery, Tri-Services General Hospital, National Defense Medical Center, Taipei 114, Taiwan; 2 Division of Hematology/Oncology, Tri-Service General Hospital, National Defense Medical Center, Taipei 114, Taiwan

**Keywords:** breast cancer, diet, dietary pattern, Mediterranean diet, paleolithic diet, prevention, western diet

## Abstract

Breast cancer is a significant public health problem globally and prevention strategies have become of great interest as its incidence rises. Exploring the connection between dietary patterns and the reduction of breast cancer risk is considered a promising approach. High levels of fiber, phytochemicals, a good antioxidant profile, and a composition of advantageous fatty acids are characteristics of healthy dietary programs such as the Mediterranean diet. This review summarized and discussed the active compounds that are considered important in preventing breast cancer, including dietary components from recent related reports. These include polyunsaturated fatty acids, fiber, phytochemicals, and alcohol. Although the exact mechanism for preventing breast cancer using these dietary factors is not well understood, the combination of all the elements in a healthy diet plays a role in reducing breast cancer risk. Considering the elevated probability of breast cancer relapse and mortality, it is crucial to investigate the correlation between a nutritious dietary pattern and breast cancer, while identifying bioactive components that have the potential to mitigate the risk of breast cancer incidence.

## 1. Introduction

Breast cancer is a major global health issue, representing the most common type of cancer and the leading cause of cancer deaths in women, with the highest number of recorded cases of all cancers. In 2020, an estimated 2.26 million new cases of breast cancer were reported worldwide [1]. An Australian study estimated that the prevalence of metastatic breast cancer was 3 or 4 times that of breast cancer deaths [2]. Siegel et al. [3], using data from the North American Association of Central Cancer Registries (NAACCR), estimated that 300,590 new breast cancer cases would be diagnosed in the United States in 2023 and 43,700 deaths would occur.

Although the prevalence of breast cancer is high, the precise cause is not fully understood. Researchers are still working to identify specific factors that contribute to breast cancer development and to devise new treatments and prevention strategies. Breast cancer is found to be associated with an array of genetic, demographic, environmental, reproductive, and lifestyle factors [4]. First, the majority of breast cancer patients are female; only about 1 of 100 breast cancer patients are male [3,5,6]. Age and race/ethnicity are also known risk factors. Breast cancer is found mainly in middle-aged and older women. The median age of onset of breast cancer is 62, with a slightly lower age of onset observed among Black women in comparison to White women [7]. Approximately 5%–10% of breast cancer cases can be attributed to genetic factors, specifically mutations in BRCA1 and BRCA2 [8,9]. Breast cancer is correlated with various reproductive factors, such as nulliparity, early age at menarche, delayed age at first live birth, advanced age at menopause, extended duration between menarche and initial pregnancy, multiple abortions, initial pregnancy after 35 years of age, and minimal or absent breastfeeding [10,11]. Elevated risk of breast cancer is associated with being overweight or obese, inadequate physical activity, tobacco usage, and alcohol intake [12,13]. Long-term exposure to harmful environmental factors may also increase the risk of breast cancer. Ionizing radiation has long been known as a risk factor for breast cancer [14]. More recently, the deleterious effects of environmental hormones (endocrine disruptors) have also gained attention. These substances include but are not limited to bisphenol A, dichlorodiphenyltrichloroethane, polychlorinated biphenyls, and phthalate plasticizers, which disrupt the hypothalamic-pituitary-gonadal axis and potentially cause reproductive disorders or cancer [15,16,17].

This review presents a summary of research papers investigating the connections between breast cancer and dietary patterns, as well as an overview of substances linked to breast cancer prevention and an exploration of current ongoing research in this area.

## 2. Methods

The present study is a narrative review that aimed to investigate the relationship between breast cancer and dietary patterns. To ensure the rigor of this review, a systematic literature search was conducted in PubMed using the keywords “diet pattern” and “breast cancer” to identify the prevalent dietary patterns across different regions of the world. The inclusion criteria for the review were limited to peer-reviewed articles published in English and published after 2017. Subsequently, a secondary search was conducted using the keywords “breast cancer” and the specific ingredient in the identified diet patterns to explore the relationship between breast cancer and individual dietary components. The inclusion criteria for this secondary search were also limited to peer-reviewed articles published in English, but not time-frame limited. The methodology employed in this review aims to provide a comprehensive and reliable synthesis of the existing literature on the relationship between dietary patterns and breast cancer.

## 3. Results

### 3.1. Western Dietary Pattern and Breast Cancer

The Western dietary pattern is characterized by a high intake of certain ingredients shown to be detrimental to health, including refined grains, excess sugar, saturated and trans fats, and high consumption of red and processed meats [18,19,20,21].

Refined grains are grains that have had the outer bran and germ removed during processing. This process removes much of the fiber, vitamins, and minerals that are the sources of nutrients found in whole grains, making refined grains less nutritious. Eating a diet high in refined grains has been linked to several health issues. One of the main concerns is that refined grains are often low in fiber, which can lead to constipation, and can make it more difficult to maintain a healthy weight [22,23,24]. They are also quickly digested, leading to a rapid increase in blood sugar and insulin levels [25,26]. This disruption in glucose metabolism contributes to the development of type 2 diabetes and other cardiometabolic disorders [27,28,29]. Although refined grains used in manufactured food products such as cereals, breads, and crackers are often fortified with synthetic vitamins and minerals, they still lack the naturally-occurring nutrients of whole grains and are consequently less nutritious. In a study by Mey et al. [22], a comparison was made between the effects of a diet rich in whole grains and a micronutrient-matched refined grain diet. The findings of this study revealed that the consumption of whole grains leads to a favorable enhancement of protein turnover and yields better health outcomes compared with the consumption of refined grains. While the inverse association was limited to case–control studies, Xiao et al. [30] reported a pooled relative risk of breast cancer to be 0.84 (95% confidence interval: 0.74–0.96, *p* = 0.009) among individuals with a high intake of whole grains compared to those with low intake. Mourouti et al. [31] also reported that the adjusted odds ratio of breast cancer in females eating whole grains more than seven times/week was 0.49 (95% confidence interval, 0.29–0.82) compared with those who never ate whole grains or did so with less frequency.

Excess added sugar is another prominent characteristic of the Western diet, which can be found in a variety of sources such as sweetened beverages and sweetened cereals, along with cookies, cakes, and candies. These added sugars contribute a significant amount of calories to the diet, with beverages alone accounting for 47% of added sugar intake [32]. In addition to non-communicable chronic diseases, it has been demonstrated that excessive sugar consumption is linked to an increased risk of breast cancer [33,34,35,36,37].

The Western diet is characterized by an elevated consumption of detrimental fats, comprising excessive quantities of trans and saturated fats, abundant levels of omega-6 polyunsaturated fatty acids (PUFA), and insufficient levels of omega-3 PUFA [38,39,40]. Of particular concern is the deleterious effect of a distorted omega-6/omega-3 ratio of 20:1, which is known to trigger metabolic complications, notably inflammatory processes [41,42,43]. Foods derived from animals, such as red meat, butter, cheese, and cream contain saturated fats. Diets with high saturated fat are linked to increased levels of very low-density lipoprotein [44,45], a higher risk of cardiovascular diseases [46], and non-alcoholic steatohepatitis [47]. A high-fat diet is shown to induce obesity, chronic inflammation, gut microbiota dysbiosis, and cancer [48,49]. The fat content of meat and dairy products from ruminant animals may contain up to 6% of naturally occurring trans fats. However, in the case of manufactured products such as crackers, cookies, and fried foods, the presence of industrial trans fats may account for up to 60% of the fat content due to the utilization of partially hydrogenated vegetable oils [50]. Unlike the protective role of unsaturated acid toward inflammation and endoplasmic reticulum stress, industrial trans fats stimulate these two deleterious processes [51]. The European Prospective Investigation into Cancer and Nutrition (EPIC) undertook a study to assess the relationship between trans fat consumption and the risk of breast cancer [52]. The findings indicated that an increased intake of industrial trans fats was correlated with a heightened risk of breast cancer in the multivariable-adjusted model (hazard ratio = 1.14, 95% CI 1.06–1.23; *p* = 0.001).

The typical Western diet is also characteristically low in consumption of fresh fruits and vegetables [19,53]. These foods are rich in phytonutrients and fiber, which help to regulate digestion/metabolism and blood sugar levels and reduce the risk of breast cancer. Fruits and vegetables are also good sources of antioxidants, which help to reduce inflammation and protect the body against cancer.

Overall, the Western dietary pattern may be high in calories and low in nutrients, making it easy for individuals to consume more calories than the body needs, leading to weight gain and increasing the risk of obesity. Being overweight or obese is a known risk factor for breast cancer [54]. Inflammation is also stimulated by the Western diet and is found to increase the risk of breast cancer [55]. Additionally, a case–control study revealed that women eating a Western diet had a higher chance of developing breast cancer (OR = 2.13, 95% CI = 1.09–4.15) [56].

### 3.2. Mediterranean Dietary Pattern and Breast Cancer

The conventional Mediterranean dietary pattern has attracted considerable interest following observations dating back to the 1960s, which revealed that populations residing in Greece and Italy had lower mortality rates associated with cardiovascular disease than Northern European populations or the United States [57]. This divergence was ascribed to variations in eating patterns, which prompted more research into the potential health advantages of the Mediterranean diet.

The Mediterranean diet is distinguished by a high intake of fresh fruit, vegetables, nuts, legumes, unrefined cereal grains, and olive oil, in addition to moderate consumption of fish and dairy products [58]. It is characterized by a lower intake of red meats than the Western diet, and the moderate utilization of ethanol, primarily in the form of red wine during primary meals. This dietary pattern has been linked to numerous potential health benefits, such as a decreased risk of cardiovascular disease and potentially certain types of cancer [58,59,60].

Breast cancer incidence has decreased as a result of the Mediterranean diet’s preventative effect against the disease’s development [61,62,63]. According to a recent case–control study, adherence to a Mediterranean diet that is abundant in fruits, vegetables, fish, and olive oil may lower the likelihood of breast cancer in pre- and post-menopausal women [64]. This favorable outcome is ascribed to the regular consumption of food-sourced fiber, flavonoids, and antioxidants, which are hypothesized to reduce estrogen levels, elevate sex hormone levels, neutralize free radicals, protect DNA from harm, and diminish oxidative stress [64]. Details of dietary components are shown below.

#### 3.2.1. Fruits, Vegetables, and Plant-Based Foods

The plant-based foods in the Mediterranean diet are typically fresh and minimally processed [65]. Evidence from several studies suggests that consuming fruits and vegetables, particularly cruciferous vegetables, may reduce the risk of breast cancer [66,67,68]. Numerous naturally occurring substances have been identified as potential cancer chemopreventive agents, displaying properties such as anti-inflammatory, antiproliferative, anti-metastatic, anti-angiogenic, and apoptotic effects [69,70,71,72]. Furthermore, the consumption of certain naturally derived dietary products at high levels may decrease the likelihood of tumor recurrence and enhance survival in breast cancer patients [73]. Many nutritional natural products found in the Mediterranean area, including pomegranate [74,75], edible mushrooms [76,77], marine algae [78,79], curcumin [80,81], whole-grain cereals [82,83], citrus fruit [84,85], grapes [86,87], and cucumber [88,89], have been shown in experimental studies to disrupt the development and progression of cancer via the application of anticancer or antineoplastic activity.

Plant-based foods contain phytochemicals shown to have health benefits that may include reducing the risk of breast cancer [90,91,92,93]. These plant-based chemicals can be found in a broad range of plant-based foods, including vegetables, fruits, grains, and nuts. Phytochemicals include a wide range of specific chemicals, including carotenoids, flavonoids, terpenes, stanols, phytoestrogens, and phenolic acids. Carotenoids found in red, orange, and yellow fruits, dark leafy vegetables, and seaweed are believed to have strong cancer-fighting properties [94]. Flavonoids found in chocolate, fruits, fungi, tea, vegetables, and wine may help prevent cancer cell growth [95]. Terpenes, a class of organic compounds found in citrus fruits, may exhibit anticancer properties by slowing the growth of cancer cells and have antiviral effects [95]. Stanols, a class of compounds found in grains, legumes, and nuts have anti-cancer abilities by affecting cell metabolism, the immune system, and cell membrane organization [96]. Phytoestrogens, including those present in berries, garlic, grapes, plums, soybeans, and tofu, have been associated with breast cancer prevention through the inhibition of local estrogen synthesis and the induction of epigenetic modifications [97]. Phenolic acids, which are present in cereals, coffee, fruits, herbs, legumes, nuts, oilseeds (peanuts, olives), and vegetables are known to have anti-inflammatory effects and may help prevent cellular damage resulting from oxidative reactions [98].

Numerous epidemiological studies have suggested that consuming fruits and vegetables, particularly cruciferous vegetables, can reduce the risk of various types of cancer, including breast cancer [66,67,68]. Several natural compounds have been identified as potential cancer chemopreventive agents due to their ability to prevent, reverse, slow, or suppress carcinogenic activities. These compounds have been shown to exhibit antiangiogenic, anti-inflammatory, antimetastatic, antiproliferative, and apoptotic properties in various types of cancer, such as breast cancer [66,69,70,71,72,99]. Furthermore, a high intake of certain natural dietary products may reduce the risk of breast cancer recurrence and improve survival [73]. Extensive experimental studies have indicated that many nutritional natural products, including citrus fruit, curcumin, grapes, edible macrofungi, marine macro- and micro-algae, mango, pomegranate, teas, spices, and whole grain cereals as mentioned earlier, are capable of interfering with the development and progression of cancer by exerting anticancer activity [100].

A dietary pattern rich in fruits, soy, and vegetables was associated with a reduced risk of postmenopausal breast cancer in Chinese women, particularly in those with ER- subtypes [101]. This Mediterranean-style diet, observed in an Asian population, is defined by the consumption of plentiful plant-based foods, which is a fundamental component of the traditional Mediterranean diet known for its potential anticancer, anti-inflammatory, and antioxidant effects [102].

#### 3.2.2. Carotenoids

Carotenoids are natural pigments that provide an orange–red color to many fruits and vegetables, such as carrots, cantaloupe, melons, papaya, pumpkin, squash, sweet potatoes, tangerines, and tomatoes. Several studies suggest that carotenoids are bioactive compounds found in various edible plants that have the potential to prevent cancer.

Carotenoids can be categorized into two principal classes, namely, carotenes and xanthophylls [103]. Xanthophylls cannot be converted into vitamin A precursors and are mainly used in photosynthesis [103,104]. Carotenes, on the other hand, are all vitamin A precursors and are shown to display antioxidant activity. Over 95% of the total carotenoids in human blood are composed of six carotenes, namely, α-carotene, β-carotene, β-cryptoxanthin, lycopene, lutein, and zeaxanthin [105]. Peraita-Costa et al. [106] conducted a review of 28 epidemiological studies to summarize the association between breast cancer and carotenoids. The authors observed an inverse association between carotenoid intake, particularly β-carotene, and the risk of breast cancer. Other studies have reported that circulating carotenoids, including α-carotene, β-carotene, β-cryptoxanthin, lutein, lycopene, and zeaxanthin, are inversely correlated with the risk of breast cancer [106,107,108]. Thus, these carotenes not only provide remarkable antioxidant properties [109] but also inhibit tumor growth and invasiveness and induce apoptosis [110].

Fucoxanthin is a prominent carotenoid pigment, which accounts for over 10% of the total estimated carotenoid production in nature, particularly in algae. [111,112,113]. It is known for its anti-cancer, anti-inflammatory, and anti-oxidant properties [114]. Seaweed is a traditional source of fucoxanthin in East Asian countries such as Japan, where it has been used medicinally for centuries. Studies have suggested that fucoxanthin exhibits anti-inflammatory effects in cancer prevention and treatment [111,112,113,114]. Moreover, emerging research suggests that compounds such as astaxanthin, curcumin, hydroxytyrosol, oleuropein, resveratrol, and spermidine may provide protection by acting as antioxidants and promoting the induction of mitophagy mediators [115].

#### 3.2.3. Polyphenols

Polyphenols are a class of bioactive compounds present in various plant-based foods that are categorized into flavonoids, polyphenolic amides, phenolic acids, and other polyphenols. Flavonoids constitute approximately 60% of the polyphenol content in nature, while phenolic acids account for about 30% [116,117,118]. Flavonoids found in legumes, fruits, vegetables, green tea, and red wine have antioxidant and anti-inflammatory properties. They are further subdivided into six classes: anthocyanins, flavanols, flavonols, flavones, flavanones, and isoflavones [119].

Anthocyanins are colored pigments abundant in the most colorful fruits and vegetables, including berries (such as raspberries, blueberries, elderberries, black currants, and strawberries) as well as purple carrots, red cabbage, eggplants, red grapes, black plums, black cherries, and blood oranges [120,121,122]. Due to the large presence of these foods in the diet, anthocyanins are the most prominent dietary flavonoid in the Mediterranean diet. Anthocyanins possess antioxidant and antimicrobial properties and have been found to inhibit the abnormally activated ERK1/2 and Akt/mTOR signaling pathways in breast cancer cells [123]. They also promote the motility and invasion of cancer cells. In addition, a separate study demonstrated that anthocyanins effectively suppress the activation of Akt and PLCγ-1 and inhibit cell motility and invasion [58].

Flavonols are the most ubiquitous flavonoids found in fruits and leafy vegetables. Quercetin and kaempferol are the most prominent flavonols, and other abundant food sources include onions, curly kale, leeks, and broccoli. Quercetin induces G2/M arrest and apoptosis, and inhibits cell proliferation in breast cancer T47D cells [124]. A different investigation revealed that the compounds quercetin and thymoquinone were able to upregulate P53 genes and DNA damage markers, resulting in significant cytotoxicity in breast cancer cells [125]. Those investigators also found that the expression of DNA repair genes was suppressed in several studied cancer cell lines.

Flavones consist chiefly of the glycosides of luteolin and apigenin. They are rich in tomatoes, eggplant, olives, thyme, peppermint, dill weed, oregano, parsley, and rosemary. Luteolin was found to suppress the expression of key transcription factors associated with stemness, as well as the expression of ABCG2, aldehyde dehydrogenase 1, CD44, Cripto-1, heme oxygenase 1, and Nrf2, all of which play crucial roles in sustaining the cancer stem-cell phenotype of breast cancer [126]. In another study, it was demonstrated in both in-vitro and xenograft animal models that the combination of luteolin and indole-3-carbinol had a synergistic effect in restricting ERα-positive breast cancer by inhibiting the estrogen receptor alpha and the cyclin-dependent kinase 4/6 pathway [127].

Romanos-Nanclares et al. [128] investigated the association between breast cancer and phenolic acid-derived compounds in a Mediterranean cohort and noticed that 40% of the total amount of polyphenols consumed by the study population was made up of phenolic acids, with a median daily consumption of 260 mg (interquartile range: 70 to 376 mg). They also reported a decreased incidence of breast cancer was seen in postmenopausal women who consumed more hydroxycinnamic acids, notably the chlorogenic acids present in coffee, fruits, and vegetables.

Chlorogenic acid, which can be found in apples and coffee, showed the ability to inhibit oxidative damage, mitochondrial dysfunction, and epithelial–mesenchymal transition and invasion in breast cancer [129,130,131]. These inhibitory properties of chlorogenic acid were achieved by inducing apoptosis through Bax, Bcl-2, caspase-3, and p53 signaling pathways [132,133].

Although these phytochemicals have demonstrated significant effects in cancer prevention, they are not the only players in the scenario. Several other compounds play a role in prevention strategies.

#### 3.2.4. Digestive Fiber

A class of polysaccharides called digestible fiber is present in the intermediate lamella and cell walls of terrestrial plants. Consuming fruits and vegetables provide fiber. Short-chain fatty acids (SCFAs), which are tiny organic carboxylic acids with 1 to 6 carbon atoms, are created during their fermentation by bacteria in the colon. The most prevalent types produced in the human gut are acetate, propionate, and butyrate, in an approximated 3:1:1 ratio [134]. Other SCFAs produced in smaller amounts include formate, isobutyrate, 2-methylbutanoate, valerate, and isovalerate. The relative amounts of different SCFAs vary depending on the type of fiber consumed, the individual’s gut microbiota, and their overall health status.

To create energy sources for survival, anaerobic microorganisms ferment digestive fiber to generate SCFAs. However, the released SCFAs can also be used as fuel for colonic mucosal epithelial cells and help to maintain physiological functions in various host tissues as they are transported through the bloodstream [135]. The systemic availability of SCFAs varies. A healthy human study utilizing stable isotopes found that acetate, propionate, and butyrate had systemic availabilities of 36%, 9%, and 2%, respectively [136], indicating that these SCFAs enter the bloodstream in varying amounts and affect different parts of the body. Except for serving as fuel, SCFAs were shown to modulate cell function through binding to G-protein coupled receptors and modulating histone deacetylation [137].

Newer research suggests that the effectiveness of cancer-treating chemotherapy, immunotherapy, and radiotherapy may be affected by SCFAs [138]. The immunomodulatory properties of SCFA may be the key factor in altering the amount of immune-suppressing Tregs and tumor-killing CD4+ and CD8+ T cells [139,140,141]. Furthermore, histone deacetylase inhibitors (HDACs), including butyrate, have been associated with regulating cell cycle and proliferation and tested as anti-cancer agents as the other HDACs inhibitors [142,143,144]. Various treatment modalities have distinct effects on the gut microbiota, which may affect the interaction with SCFAs in diverse ways.

Pectin derived from fruits and olives is a widely studied digestive fiber. In addition to being fermented into SCFAs, pectin also lowers levels of low-density lipoprotein (LDL) cholesterol [145,146]. Pectin may also help prevent the reabsorption of bile salt micelles, although the exact mechanism is not fully understood [146,147]. Studies have found that pectin and its derivatives may suppress cancer cell growth and stimulate apoptosis [148,149]. When combined with other plant compounds, pectin has demonstrated the ability to decrease the invasive potential of human breast cancer cells. Additionally, pectin may prevent the development of breast cancer in mice by decreasing angiogenesis. [148,150]. Pectin may also inhibit the synthesis of urokinase-type plasminogen activator and the urokinase receptor, which are involved in the migration and invasion of cancer cells [148,151]. Pectin has also been shown to activate macrophages and affect signaling pathways involved in inflammation [152]. In addition, pectin and, in particular, apple pectin has been shown to inhibit the growth of breast cancer cells and reduce the expression of a lectin called Gal-3, which is involved in cell adhesion, cell cycles, and cell death [148,153]. Pectin has also been shown to cause oxidative and strand-breaking DNA damage in breast cancer cells, slowing their proliferation [148,154,155].

#### 3.2.5. Olive Oil

The primary source of calories and fat in the traditional Mediterranean diet is olive oil, limited to extra virgin olive oil. [156]. Olive oil’s major fatty acid, oleic acid, an n-9 monounsaturated fatty acid (MUFA), accounts for 55–83% of the oil’s total fatty acid concentration [157]. Other substances in olive oil include saturated fatty acid, polyunsaturated fatty acids (PUFA), vitamins, and polyphenols.

The most prevalent fatty acid in healthy persons is oleic acid. It is present in plasma, adipocytes, and cell membranes [158]. It has been demonstrated that eating a diet high in oleic acid helps those with inflammatory conditions by activating numerous immunological-competent cell pathways [159]. The effects of oleic acid in breast cancer are still controversial. It had been shown that oleic acid had inhibitory effects on low metastatic cancer cells while selectively promoting cell proliferation and migration in highly metastatic cancer cells [160]. In a coculture system with triple-negative breast cancer cells, lipid peroxidation and ferroptosis, a non-apoptosis cell death, was inhibited by oleic acid secreted from adipocytes [161]. Oleic acid also increases cancer cell apoptosis, intracellular caspase 3 activity, and the development of reactive oxygen species [162].

Polyunsaturated fatty acids (PUFAs) are fatty acids with long carbon chains that contain more than one double bond in their backbone. They are categorized into *n*-3 PUFAs and *n*-6 PUFAs, based on the location of the first double bond in the carbon chain [163]. In contrast to saturated or trans fatty acids, PUFAs have been regarded as advantageous for human health due to their anti-inflammatory properties [164]. In olive oil, *n*-6 PUFA linoleic acid contributes to about 3.5–21% of anti-atherosclerotic effects, and *n*-3 PUFA linolenic acid accounts for another 0–1% [165,166]. The risk of breast cancer was found to be inversely related to dietary *n*-3 polyunsaturated fatty acids [167]. Additionally, a higher ratio of *n*-3/*n*-6 PUFAs was linked to a lower risk of breast cancer [168,169]. The Western diet generally is rich in *n*-6 PUFA. Prospective studies have suggested that the consumption of *n*-6 PUFAs is not significantly linked to the risk of cancer, whereas the levels of *n*-6 PUFAs in blood have an inverse association with cancer risk. Western populations, with a greater intake of *n*-6 PUFAs, specifically linoleic acid (LA, 18:2*n*-6), and lower *n*-3 PUFA intake compared to Asian populations, have a higher incidence of breast cancer [170]. Due to the high intake of *n*-6 PUFA in the Western diet, Asian-American women have a 60% higher chance of developing breast cancer than white women [171,172].

Although the benefits of olive oil are still not clear, besides *n*-3 PUFA and *n*-9 MUFA, olive oil contains an abundance of polyphenols. The phenolic composition of extra-virgin olive oil (EVOO) is highly intricate and comprises several phenolic alcohols, such as hydroxytyrosol and tyrosol, as well as secoiridoids, oleacein, and oleocanthal esters [173,174,175]. Oleacein is known to be the principal antioxidant polyphenolic compound present in EVOO and has been attributed, at least in part, to its strong anti-inflammatory activity [176,177]. EVOO is a rich source of bioactive compounds, mainly monounsaturated fatty acids, triterpenes, and polyphenols, including phenolic alcohols (e.g., hydroxytyrosol), flavonoids (e.g., luteolin), lignans (e.g., pinoresinol), and secoiridoids (e.g., oleuropein and oleocanthal) [178].

#### 3.2.6. Fish

Breast cancer appears to be one of the cancer types where fish consumption appears to have a favorable impact [179]. According to a study done in the Makkah area of Saudi Arabia, those who eat a Mediterranean-style diet can reduce their risk of breast cancer by 78.9% to 92.8% by eating up to five servings of fish and shellfish per week [180]. However, Engeset et al. [181] found no proof that a person’s overall fish consumption and risk of breast cancer were inversely related. But their research demonstrated that PUFAs inhibited the epidermal growth factor receptor, which in turn decreased the growth of breast cancer.

Omega-3 fatty acids, especially eicosapentaenoic acid (EPA) and docosahexaenoic acid (DHA), which had been demonstrated to lower the risk of breast cancer, were known to be found in fish [182,183]. Monk et al. [184] reported that partial substitution of corn oil for fish oil in a high-fat diet increased the expression of a pro-apoptotic marker, Bad, and decreased anti-apoptotic Bcl-xL mediator expression. In addition, the mRNA levels of inflammatory mediators, such as IL-6, leptin, and TNFα were reduced. On the contrary, IL-10 expression was increased in subjects in a low-fat group who consumed a fish oil substitute. Another study found that n-3 PUFAs from marine sources, such as EPA and DHA, are 8 times more effective than those from plants in preventing breast cancer [185]. Thus, fish consumption that provides marine-derived n-3 PUFAs appears to reduce the risk of breast cancer.

#### 3.2.7. Alcohol Consumption

The risk of breast cancer is one of the major health concerns related to alcohol consumption. Studies have found that alcohol is a significant contributor to breast cancer. Xu et al. [186] conducted a study on adolescent mice and demonstrated that alcohol exposure had a significant impact on the latency period for tumor development. Specifically, the latency period was shortened from 18.5 to 22 weeks in the control group to 9.5 and 8.4 weeks in the experimental groups. The study also revealed that alcohol exposure initiated during adolescence led to notable changes in mammary epithelial cell proliferation, ductal growth, and terminal end bud formation in the mammary glands.

Although alcohol consumption is a risk factor for breast cancer, a J-shaped curve is often used to describe the relationship between alcohol consumption and health outcome [187,188]. Drinking alcohol within a moderate amount shows beneficial effects, but excessive drinking leads to increased all-cause mortality.

Traditionally, a small amount of wine from grapes was consumed during the meal in the Mediterranean diet. Many bioactive components discussed above were detected in the wine [189]. Another study also reported that flavonols, catechins and epicatechin, proanthocyanidins, anthocyanins, various phenolic acids, and stilbene resveratrol are found in wine [189,190,191]. These compounds are reported to have antioxidant and anti-inflammatory properties, which may help to reduce the risk of certain diseases [192]. The question remains about whether or not other hard liquids have similar protective effects as moderate wine consumption.

Numerous phenolic chemical groups, primarily flavones, flavonols, phenolic acids, and tannins, have been found in beer [193,194]. One of the phenolic compounds, tyrosol, can sometimes be as rich as in red wine [195]. Spirits, distilled alcoholic drinks, lose about 60% of their phenolic compounds after brewing [194]. However, storage in wood barrels provides additional phenolic compounds during maturation [196,197]. When comparing the health effects of wine, beer, and vodka, Krnic et al. [198] found that only red wine offered a defense against oxygen-induced oxidative stress.

## 4. Dietary Pattern and Histological and Molecular Classification of Breast Cancer

Breast cancer is a heterogeneous disease with various histological subtypes, including apocrine carcinoma, medullary carcinoma, metaplastic carcinoma, mucinous carcinoma, classic lobular carcinoma, cribriform carcinoma, neuroendocrine carcinoma, pleomorphic lobular carcinoma, tubular carcinoma, and the most common type, invasive ductal carcinoma (IDC). Breast cancer has been divided into four molecular subgroups that are well-established in clinical practice as a result of the identification of intrinsic subtypes using global gene expression profiles. These subgroups include HER2+, Luminal A, Luminal B, and Triple Negative. The evidence for the association between certain histological or intrinsic breast cancer and the dietary pattern is accumulating. Foroozani et al. [199] reported that Western dietary patterns had a higher risk of invasive ductal and lobular breast carcinomas. Dianatinasab et al. [56] studied the association between the risk of IDC and invasive lobular carcinoma (ILC) of the breast and Western diet or Mediterranean diet. They also reported that the Western diet had an increased risk of IDC and ILC. On the contrary, the Mediterranean diet was associated with a reduced risk of IDC and ILC. Castelló et al. [200] studied the association between Western and Mediterranean dietary patterns and breast cancer and reached a similar conclusion. In addition, they observed a significant positive association only between estrogen/progesterone (ER/PR)+ and HER2+ breast cancer and Western dietary patterns. A significant difference was not observed between other dietary patterns and breast cancer subtypes.

## 5. Conclusions

**Figure 1:**
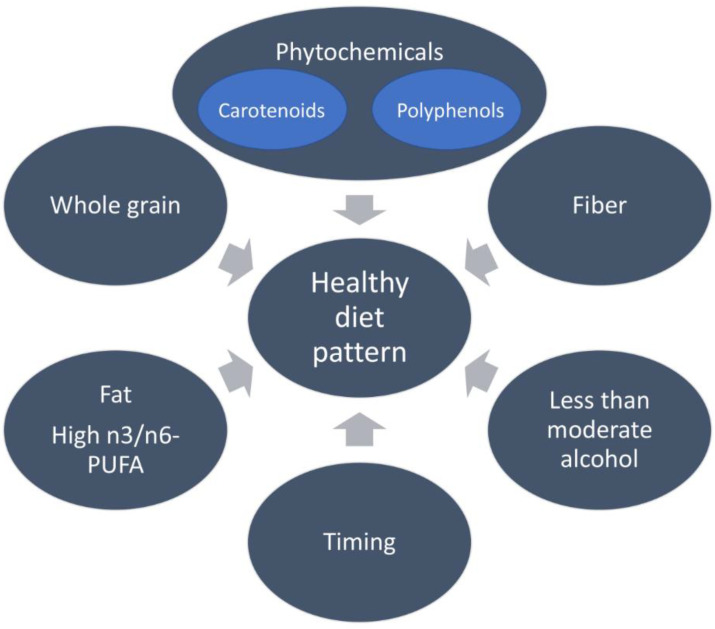
The ingredients of a healthy diet pattern. Besides providing enough nutrition, a healthy diet pattern is affected by many factors shown in the figure.

A healthy diet pattern contains various ingredients as shown in Figure 1. It is widely accepted that a diet rich in phytochemicals and fiber can have a positive impact on overall health. These bioactive compounds, found in fruits, vegetables, whole grains, and legumes, have been shown to play a role in modulating various functions within the body, and in some cases, may act in conjunction with the gut microbiome to produce health benefits. While no single food can cure or prevent disease, incorporating a dietary pattern that emphasizes these nutrient-dense foods, such as in the Mediterranean diet, is recommended for optimal health. However, a balanced diet and lifestyle, including regular physical activity and limited alcohol consumption, are also key factors in maintaining health.

## Data Availability

The data presented in this study are available on request from the corresponding author. The data are not publicly available due to restrictions concerning privacy and ethical reasons.
